# Isolation of viruses, including mollivirus, with the potential to infect *Acanthamoeba* from a Japanese warm temperate zone

**DOI:** 10.1371/journal.pone.0301185

**Published:** 2024-03-28

**Authors:** Daichi Morimoto, Naohisa Tateishi, Michiko Takahashi, Keizo Nagasaki

**Affiliations:** 1 Faculty of Science and Technology, Kochi University, Nankoku, Kochi, Japan; 2 Faculty of Agriculture and Marine Science, Kochi University, Nankoku, Kochi, Japan; 3 Kochi Medical School, Kochi University, Nankoku, Kochi, Japan; Iran University of Medical Sciences, ISLAMIC REPUBLIC OF IRAN

## Abstract

*Acanthamoeba castellanii* is infected with diverse nucleocytoplasmic large DNA viruses. Here, we report the co-isolation of 12 viral strains from marine sediments in Uranouchi Inlet, Kochi, Japan. Based on the morphological features revealed by electron microscopy, these isolates were classified into four viral groups including *Megamimiviridae*, *Molliviridae*, *Pandoraviridae*, and *Pithoviridae*. Genomic analyses indicated that these isolates showed high similarities to the known viral genomes with which they are taxonomically clustered, and their phylogenetic relationships were also supported by core gene similarities. It is noteworthy that *Molliviridae* was isolated from the marine sediments in the Japanese warm temperate zone because other strains have only been found in the subarctic region. Furthermore, this strain has 19 and 4 strain-specific genes found in *Mollivirus sibericum* and *Mollivirus kamchatka*, respectively. This study extends our knowledge about the habitat and genomic diversity of *Molliviridae*.

## Introduction

*Acanthamoeba* are free-living protists that are widely distributed in the environment. These protists incorporate bacterial cells into phagosomes via pseudopod extension. In this step, certain viruses are also incorporated into *Acanthamoeba* cells, and cell lysis occurs in some cases [1, 2]. Owing to this unique feature, several *Acanthamoeba* species, including *A*. *polyphaga* and *A*. *castellanii* have been used as hosts to isolate viruses.

*Acanthamoeba polyphaga mimivirus* is the first identified giant virus that infects *Acanthamoeba*; it was isolated in 1992 and identified in 2003 [3]. Since this discovery, several *Acanthamoeba* viruses that belong to diverse families have been isolated [1, 2]; *Asfarviridae* [4], *Marseilleviridae* [5], *Medusaviridae* [6], *Megamimiviridae* [3], *Molliviridae* [7], *Pandoraviridae* [8], and *Pithoviridae* [9, 10]. These giant viruses replicate themselves in structures known as “viral factories” built in the host cytoplasm or directly exploit the host nucleus to replicate and assemble viral progeny [11]. Thus, these viruses are also called nucleocytoplasmic large DNA viruses (NCLDVs) [12, 13].

NCLDVs are known to have a set of highly conserved genes (core genes). These core genes encode proteins involved in important cellular processes such as nucleotide synthesis, DNA replication, DNA recombination and repair, and transcription [13]. Phylogenetic analysis based on the presence or absence of these genes suggested that NCLDVs are monophyletic and represent a fourth domain of life that originated from a common ancestor [14]. Furthermore, recent metagenomic analysis predicted that horizontal gene transfer occurs between various NCLDVs and host eukaryotes [15]. Therefore, NCLDVs are important biological entities for understanding evolutionary processes and ecological networks [2, 15].

These NCLDVs universally and heterogeneously exist in marine environments [16]. For example, high proportions of unique NCLDVs are present in the polar biomes [16]. Likewise, *Molliviridae* were only isolated from the subarctic region in areas such as Siberia and Kamchatka [10, 17]. Therefore, investigating NCLDVs in different locations is essential for elucidating the marine ecosystem dynamics.

Uranouchi Inlet is a small semi-enclosed sea area located at the southeastern side of Shikoku Island, Japan. Although the existence of diverse *Mimiviridae* in the inlet was revealed [18], the *Acanthamoeba* viruses have not been isolated to date. Here, we isolated and characterized *Acanthamoeba* viruses of four families from Uranouchi Inlet, Japan.

## Materials and methods

### Culture conditions

*Acanthamoeba castellanii* Neff (ATCC 30010) was kindly provided by Prof. Masaharu Takemura. This strain was cultured in PYG medium (ATCC medium 712) supplemented with an antibiotic mixture at 26°C for a week. The antibiotic mixture contained 100 mg/L ampicillin (FUJIFILM Wako, Osaka, Japan), 100 mg/L chloramphenicol (Nacalai Tesque, Kyoto, Japan), 100 mg/L tetracycline hydrochloride (Nacalai Tesque), 100 mg/mL Neomycin (Nacalai Tesque), 1 mg/L penicillin-streptomycin solution (FUJIFILM Wako), and 25 mg/L amphotericin B (FUJIFILM Wako).

### Isolation of lytic agents causing *A*. *castellanii* cell death

Soil samples were collected in Uranouchi Inlet, Kochi Prefecture, Japan from 22 August 2019 through 30 July 2020 (S1 Fig). Permission for sampling for this study was obtained from the Japan Coast Guard and Kochi Prefecture. Up to 3 g of the sample was suspended in a 10-fold volume of distilled water, and then stirred at room temperature for 1 h. The suspended samples were incubated at 4°C until they naturally settled. Each supernatant was filtered through 5.0-μm pore size cellulose membrane (150 mm, ADVANTEC, Tokyo, Japan). An aliquot (50 μL) of filtrates was inoculated into *A*. *castellanii* cultures (250 μL) in 96-well plates (Thermo Fisher Scientific, MA, USA) and incubated for a week under the above conditions.

Each well was monitored by optical microcopy every other day. The cell lysates were diluted with distilled water; then, serial 10-fold dilutions (250 μL) were inoculated into *A*. *castellanii* (150 μL) cultures in 96-well plates. After incubation for a week, this extinction dilution procedure was conducted again with the lysates from the most diluted well.

### Verification of bacterial absence from the lysate

Five media were prepared for sterility tests using PYG medium following instructions from the National Institute for Environmental Studies (B-I, B-II, B-IV, B-V, and YT; https://mcc.nies.go.jp/02medium.html). The lysates (10 μL) were inoculated into each medium (500 μL) and incubated in 48-well plates (Thermo Fisher Scientific) at 26°C. Each well was monitored by optical microscopy every other day. Additionally, the lysates (100 μL) were filtered through 0.2-μm (ADVANTEC) and 0.1-μm (Pall Corporation, NY, USA) syringe membrane filters, respectively, and then inoculated into *A*. *castellanii* culture (150 μL) to confirm whether the filtrates retained the lytic activity.

DNA was extracted from the lysates using the phenol/chloroform/isoamyl alcohol procedure after incubation with 10% (v/v) SDS (Tokyo Chemical Industry Co., Ltd., Tokyo, Japan) and protease K (FUJIFILM Wako) for 1 h at 56°C. The extracted DNA was subjected to PCR amplification of the 16S rRNA gene using Ex Taq (TaKaRa Bio Inc., Shiga, Japan) with 27F and 1492R primers [19]. PCR conditions were as follows: initial denaturation at 94°C for 2 min, followed by 35 cycles of 94°C for 30 s, 55°C for 30 s, and 72°C for 30 s; and a final extension at 72°C for 7 min. The resultant products were confirmed by electrophoresis on a 2% agarose gel.

### Electron microscopy

After incubation for two weeks, the lysates were prepared for electron microscopy. An aliquot (35 μL) of the samples was mixed with 5 μL of 4% osmium tetroxide, and then incubated for 5 min at room temperature. The fixed samples were collected on a 0.2-μm membrane filter (25 mm), washed twice, and immersed in distilled water at 4°C overnight. The filters were immersed in 30%, 50%, 70%, 90%, and 95% ethanol every 5 min. Then, the filters were immersed in 100% ethanol three times for 20 min at room temperature and completely dried using a critical point dryer (JEOL JCPD-5). The dried samples were coated with osmium tetroxide by an osmium coater (Neoc-Pro, Meiwafosis Co., Ltd., Tokyo, Japan) and observed using field-emission scanning electron microscopy (FE-SEM; JEOL JSM-6500F).

After culturing for one week, *A*. *castellanii* (150 mL) was mixed with 20 mL of each lysate, and then incubated for 24 h under the above conditions. An aliquot (42.5 mL) of the cultures was centrifuged at 600 *g* for 10 min at 4°C. The cell pellet was washed with 0.1 M phosphate buffer (pH6.8) and suspended in the same buffer (460 μL). The samples were fixed in 25% glutaraldehyde (Nacalai Tesque) at a final concentration of 2% and incubated for 1 h at 4°C. After washing twice, the fixed samples were resuspended in 500 μL of phosphate buffer and stored at 4°C until analysis. After centrifugation at 1,100 *g* for 5 min, the samples were mixed with 1 mL of distilled water containing 1% (w/v) Agarose-S (Nippon Gene Co., Ltd., Toyama, Japan) and solidified during centrifugation again under the same conditions. The agarose blocks with *A*. *castellanii* cells were cut into 1-mm cubes and then immersed in 500 μL of phosphate buffer.

Ultrathin sections of each sample were prepared by Dr. Kenichi Yagyu as follows. After washing with the buffer, the samples were fixed with 0.1 M phosphate buffer (pH7.3) including 1% osmium tetroxide at 4°C for 1 h, followed by dehydration treatment with ethanol. The treated samples were coated with Epon 812 (TAAB Laboratories Equipment, Reading, UK), and then ultrathin sectioned using a Leica EM UC7 microtome (Leica Microsystems, Wetzlar, Germany). After double staining with uranium and lead dye, the samples were observed using transmission electron microscopy (TEM; JEOL JEM-1400Plus).

### Viral genome sequencing, assembly, and phylogenetic analyses

After culturing for one week, *A*. *castellanii* (80 mL) were infected with each viral lysate (800 μL), and then incubated for two weeks. After centrifugation at 200 *g* for 5 min, the supernatants were further centrifuged at 20,400 *g* for 20 min. The samples were resuspended in 900 μL of distilled water, and then subjected to DNA extraction using the above-mentioned method. DNA libraries were prepared using the NEBNext Ultra II FS DNA Library Prep Kit for Illumina (New England Biolabs, MA, USA) according to the manufacturer’s instructions. Library sequencing (2 × 150-bp read length; NovaSeq 6000) was performed by Rhelixa Co., Ltd. (Tokyo, Japan). After adapter trimming and quality filtering (Q30), total reads from each sample were assembled using SPAdes version 3.15.3 with default k-mer lengths [20]. Detection of the viral signal was performed using VirSorter 2.2.3 with the “—include-groups NCLDV” option [21]. ViPTree server version 3.4 was used for proteomic tree construction, gene annotation, and genomic alignment views [22]. The genomic similarity score (*S*_G_) value was set to ≥ 0.15 (viral genus level cut-off) according to a previous study [23]. Maximum likelihood analysis of the core genes coding DNA polymerase family B and VVA18 helicase was performed using the Molecular Evolutionary Genetics Analysis (MEGA) package version 11.0.13 [24].

## Results

### Isolation of lytic agents from soil samples

During the survey period, we collected 31 soil samples from sediments at multiple stations in Uranouchi Inlet (S1 Fig). Among these samples, nine inocula showed lytic activity against *A*. *castellanii* (S1 Table). After purification by the extinction dilution method, we isolated 12 lytic agents that caused *A*. *castellanii* cell death (S1 Table).

Sterility tests of lytic agents showed no increase in turbidity in the inoculated five media due to the propagation of microbial cells. Likewise, agarose gel electrophoresis did not indicate a band corresponding to the 16S rRNA gene. Furthermore, the filtered agents showed no lytic activities against *A*. *castellanii* cells. Based on these results, we concluded that lytic activities could originate from giant viruses but not microorganisms or bacterial viruses.

### Morphological features of *A*. *castellanii* viruses

**Fig 1:**
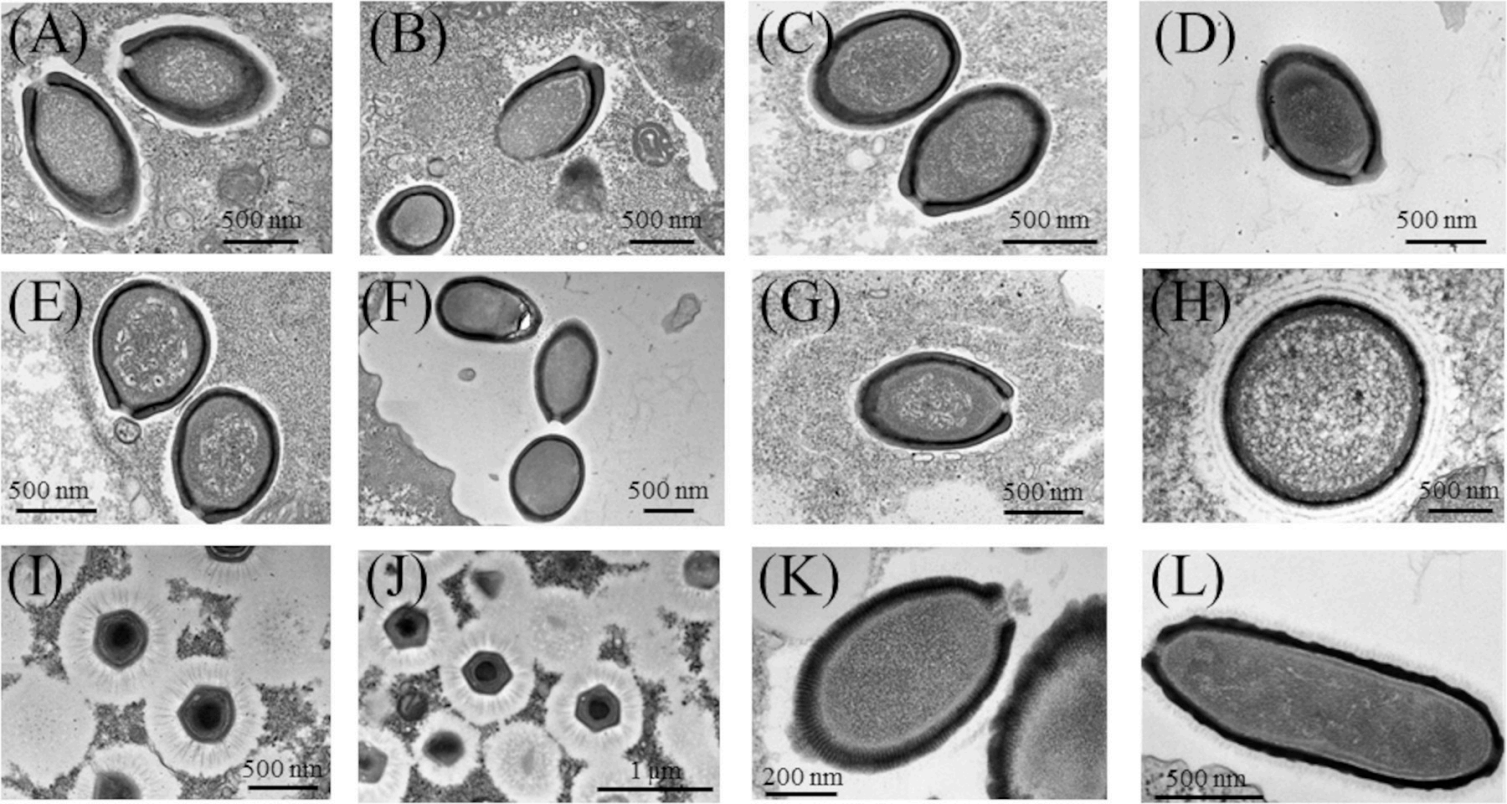
Morphological features of *A*. *castellanii* viruses isolated from marine sediments in Uranouchi Inlet. (A) Pa1-1, (B) Pa1-2, (C) Pa1-3, (D) Pa1-4, (E) Pa2-1, (F) Pa6-1, (G) Pa11-1, (H) Mo1-1, (I) Me1-1, (J) Me1-2, (K) Ce2-1, (L) Ce7-1.

**Fig 2:**
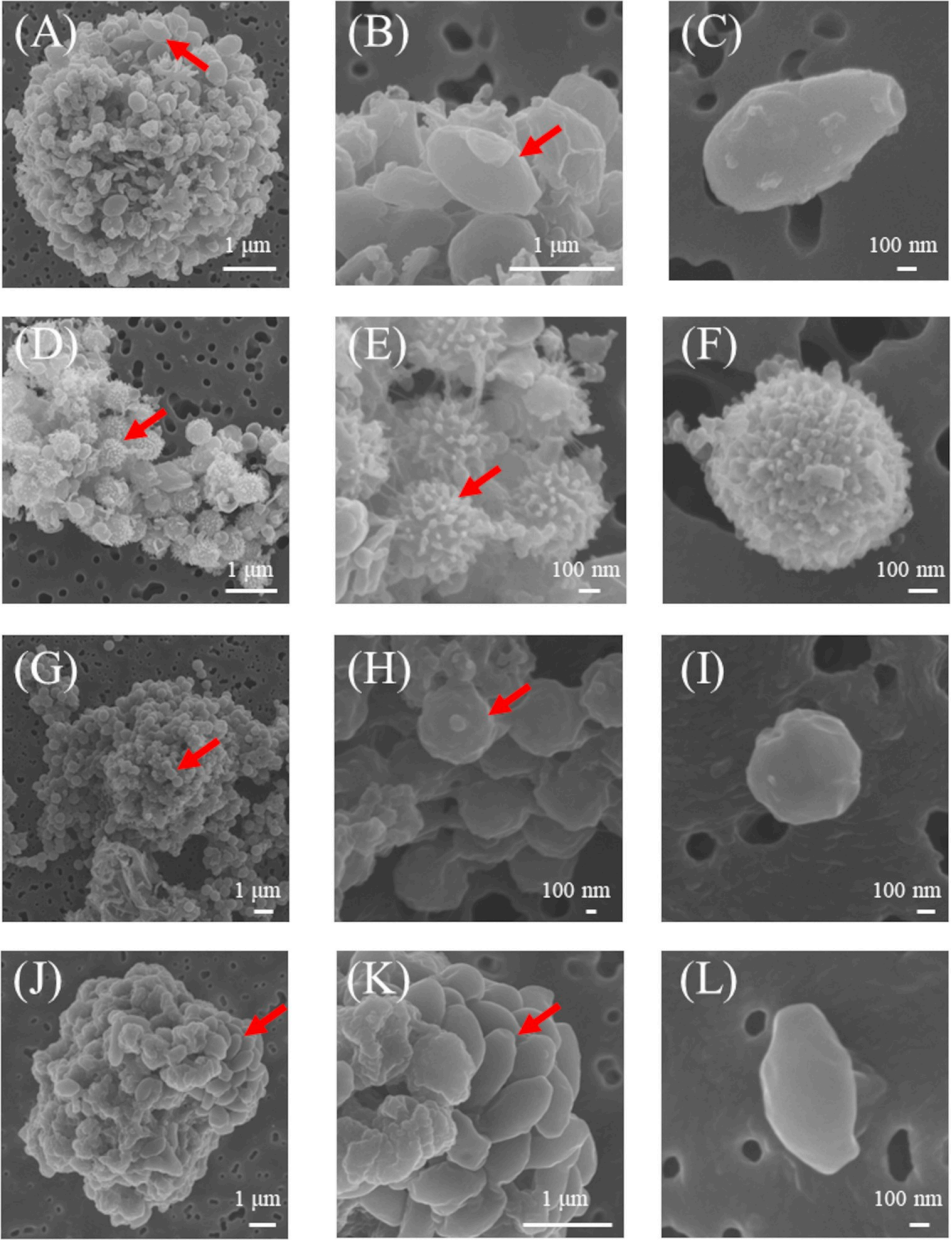
Scanning electron microscopy images of selected *A*. *castellanii* viruses isolated in this study. Left, middle, and right columns represent the images of infected cell, enlarged view of the cell surface, and viral particle, respectively. (A–C) Pa1-1, (D–F) Mo1-1, (G–I) Me1-2, (J–L) Ce7-1. Red arrows indicate viral particles adsorbed to the cell.

TEM and FE-SEM images of the lytic agents demonstrated that *A*. *castellanii* viruses isolated in this study were classified into four distinct morphotypes (Fig 1). The seven isolates showed typical features of pandoraviruses, with ovoid particles (avg. major and minor axes: 1.1 and 0.75 μm, respectively) with an apex-like aperture (Figs 1A–1G and 2A–2C) [8]. The Mo1-1 isolate had spherical particles (0.75 μm in diameter) surrounded by a hairy tegument that consisted of three layers (Figs 1H and 2D–2F), which was consistent with morphological features of known molliviruses [7]. The two isolates Me1-1 and Me1-2 also showed unique morphological features of *Megamimiviridae*, including a large capsid (avg. 0.45 μm in diameter) with fibrous structures (Figs 1I, 1J and 2G–2I) [25]. The other isolates, Ce2-1 and Ce7-1, had morphological features consistent with cedratviruses, including ovoid particles (avg. major and minor axes: 1.2 and 0.7 μm, respectively) with a cork-like structure at both ends (Figs 1K, 1L and 2J–2L) [9].

### Genome analysis of *A*. *castellanii* viruses

**Fig 3:**
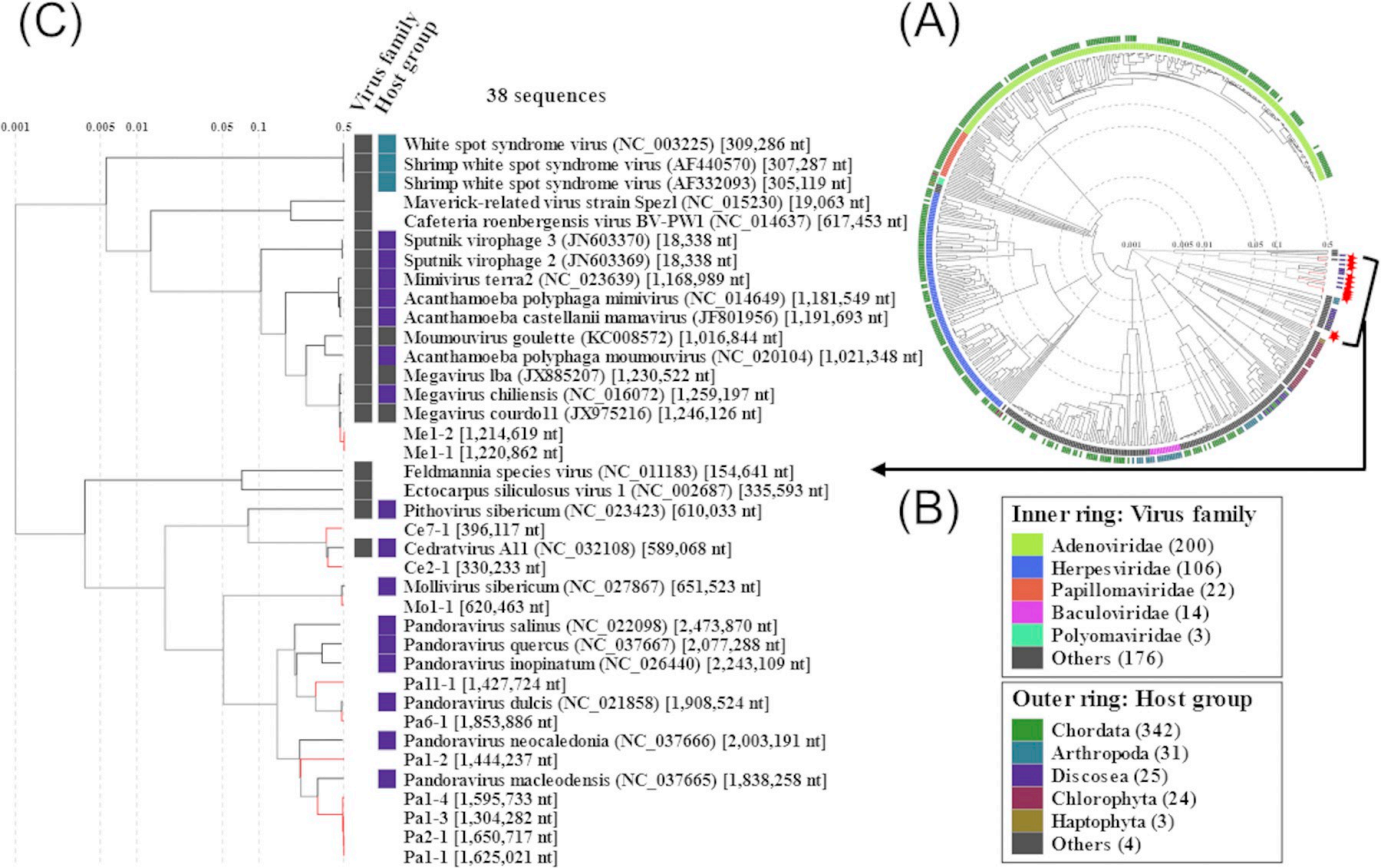
Proteomic tree of 12 *A*. *castellanii* viral genomes isolated in this study and 533 related eukaryotic dsDNA viruses. (A) Whole proteomic tree, including 534 related eukaryotic dsDNA viruses, generated by ViPTree server version 3.5. The dendrogram represents the proteome-wide similarity relationships among the 12 *A*. *castellanii* viruses isolated in this study (red branches) and reference viral genomes (black branches). Branch lengths are shown on a logarithmic scale from the root of the entire tree. (B) Inner and outer rings that are outside the dendrogram represent viral family classifications and taxonomic groups of known hosts, respectively. (C) Enlarged view of the proteomic tree that includes the viruses isolated in this study.

To reveal the genomic features, we next sequenced and assembled 12 genomes of *A*. *castellanii* viruses (≥ 10 kb) isolated from Uranouchi Inlet. *Acanthamoeba castellanii* viruses were largely classified into four groups using a viral proteomic tree [26] based on their genome similarity scores derived from tBLASTx scores (Fig 3) [22].

**Fig 4:**
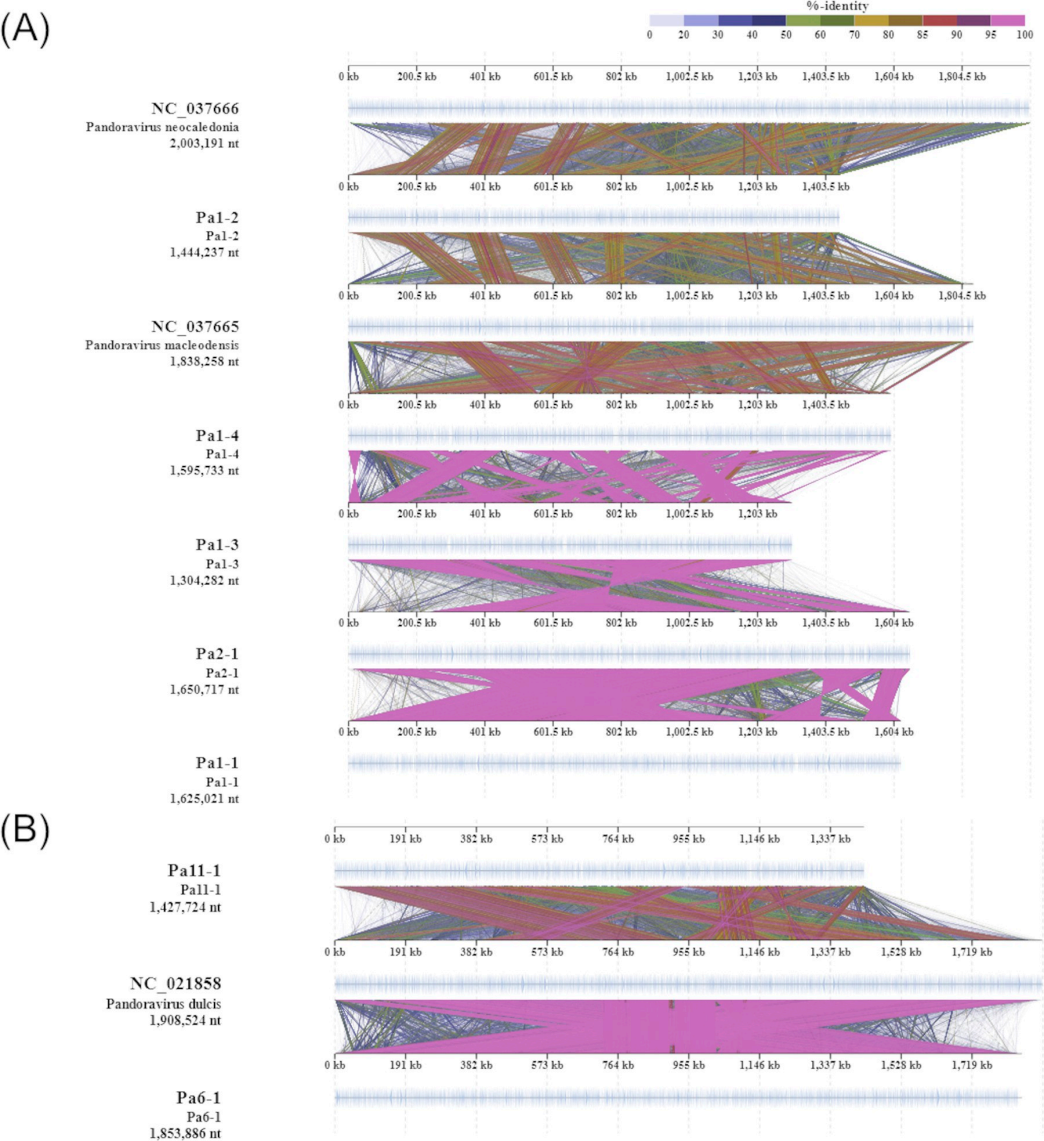
Genome map of pandoraviruses isolated in this study. (A) *Pandoravirus macleodensis* relatives, (B) *P*. *dulcis* relatives. All alignments are represented by colored lines that show the tBLASTx percent identities between two viral genomes.

The seven pandoravirus genomes ranged from 1,304,282 to 1,853,886 bp and contained 847 to 1,109 predicted protein-coding genes (S2 Table). Of these, four pandoraviruses Pa1-1, Pa1-3, Pa1-4, and Pa2-1, showed high sequence similarity with *Pandoravirus macleodensis* [27] and each other (Fig 4A). Likewise, the genome sequences of the isolates Pa6-1 and Pa11-1 were similar to that of *P*. *dulcis* (Fig 4B) [8]. Also, pandoravirus Pa1-2 showed high sequence similarity with *Pandoravirus neocaledonia* [27]. Consistent with these results, phylogenetic analysis of the DNA polymerase β gene also showed that these viruses were closely related to known pandoraviruses (S2 Fig).

**Fig 5:**
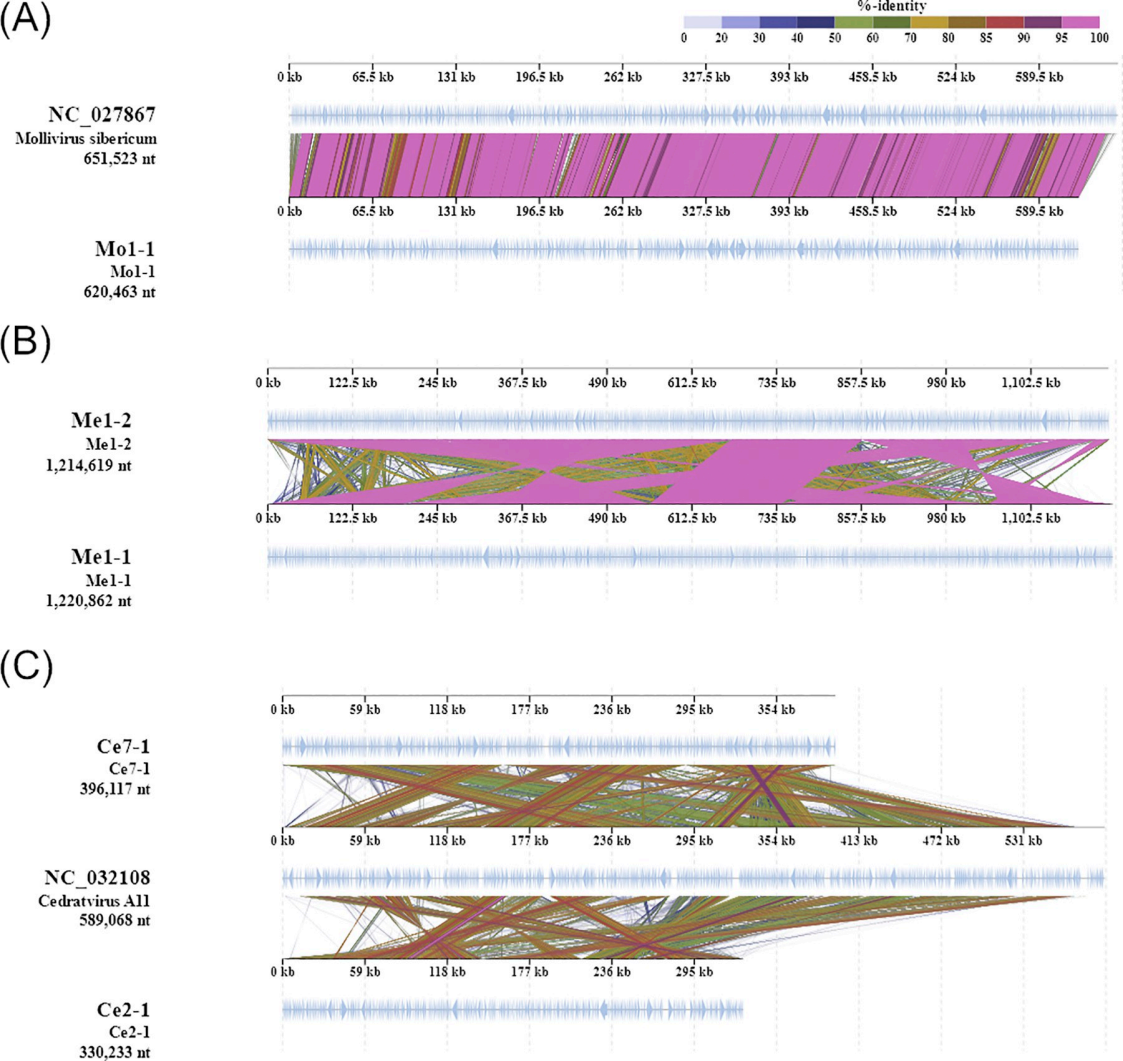
Genome map of mollivirus, megaviruses, and cedratviruses isolated in this study. (A) *Mollivirus sibericum* and a relative, (B) megavirus relatives, (C) *Cedratvirus A11* and relatives. All alignments are represented by colored lines that show the tBLASTx percent identities between two viral genomes.

Mollivirus Mo1-1 had 620,463-bp genome containing 520 putative protein-coding genes (S2 Table). This viral strain exhibited high sequence similarity with *M*. *sibericum* (Fig 5A) [10]. Additionally, phylogenetic trees of DNA polymerase β and VVA18 helicase genes indicated that this isolate is closely related to molliviruses such as *M*. *sibericum* and *M*. *kamchatka* (S2 and S3 Figs). In coincidence with this result, this strain has unique genes found in *M*. *sibericum* and *M*. *kamchatka* (S3 Table).

The *Megamimiviridae* genomes isolated in this study ranged in size from 1,214,619 to 1,220,862 bp and contained 1,089 to 1,095 predicted protein-coding genes (S2 Table). Two isolates, Me1-1 and Me1-2, displayed high sequence similarities with *Megavirus courdo11* [28], *M*. *Iba* [29], and *M*. *chiliensis* [30] (Fig 5B). The core genes of these isolates formed sister clades with megaviruses but not mimiviruses (S2 and S3 Figs).

Cedratviruses Ce2-1 and Ce7-1 had 330,233 and 396,117-bp genomes encoding 360 and 445 putative protein-coding genes, respectively (S2 Table). Both isolates exhibited high sequence similarity with *Cedratvirus A11* [9] (Fig 5C), which was also consistent with morphological features. This phylogenetic relationship was supported by the VVA18 helicase gene maximum-likelihood tree (S3 Fig).

## Discussion

In this study, we isolated the giant viruses infecting *A*. *castellanii* from marine sediments. Our results indicated that four phylogenetically distinct viral groups (*Megamimiviridae*, *Molliviridae*, *Pandoraviridae*, and *Pithoviridae*) coexist in Uranouchi Inlet, Kochi, Japan. In particular, sampling station 1, which is located at the closed-off section of the inlet, was a “hot spot” where diverse giant viruses coexisted (S1 Fig and S1 Table). However, we could not isolate giant viruses closely related to *Asfarviridae* [4], *Marseilleviridae* [5], and *Medusaviridae* [6].

All giant viruses isolated in this study showed high similarities to known viral genomes taxonomically clustered together (Figs 3–5). However, it is worth noting that mollivirus Mo1-1 was isolated from the sediment in Uranouchi Inlet. To date, *Molliviridae* has only been isolated from the subarctic region, such as from the permafrost layer and Russian riverbank [7, 17]. Therefore, this is the first report on the existence of *Molliviridae* in a warm temperate zone and endorses that they are not extinct from the current environment [17].

In the *M*. *kamchatka* genome, 96% of the encoded proteins were highly conserved compared with those of *M*. *sibericum* which was in a dormant state for 30,000 years; this indicates that most of these proteins contribute to viral fitness [17]. The highly conserved genome of Mo1-1 also supported the importance of these proteins for the mollivirus lifecycle (Fig 5A). Meanwhile, the Mo1-1 genome contains not only unique genes found in the *M*. *sibericum* genome, but also *M*. *kamchatka*-specific genes [17] (S3 Table). Further studies are needed to elucidate acquisition/loss events of these genes and their contribution to viral fitness for each *Molliviridae* strains.

In conclusion, we revealed the diversity and genomic features of *A*. *castellanii* viruses in Uranouchi Inlet, Japan. These findings expand our current knowledge regarding *Molliviridae* habitat and genomic differences among the strains. The results of this study will provide an opportunity to better understand the evolution and diversity of *Molliviridae* if they are isolated from a wide range of climatic zones in the future.

## Supporting information

S1 Fig Sampling sites in Uranouchi Inlet, Kochi, Japan. Soil samples were collected at each station (St) from 22 August 2019 through July 2020. The numbers in brackets represent those of isolated *A*. *castellanii* viruses. The map was created by editing the map vector provided by Geospatial Information Authority of Japan.
(TIF)

S2 Fig Maximum-likelihood tree of DNA polymerase β genes. The tree contains the protein sequences encoded in *Acanthamoeba* viruses, including the pandoraviruses, molliviruses, and megaviruses isolated in this study. The scale bar represents the estimated substitution number of amino acids per site. Numbers close to the nodes indicate bootstrap values above 75%.
(TIF)

S3 Fig Maximum-likelihood tree of VVA18 helicase genes. The tree contains the protein sequences encoded in *Acanthamoeba* viruses, including the cedrativiruses, molliviruses, and megaviruses isolated in this study. The scale bar represents the estimated substitution number of amino acids per site. Numbers close to the nodes indicate bootstrap values above 75%.
(TIF)

S1 Table Summary of viruses isolated from the sediment in Uranouchi Inlet. Each viral strain was named according to viral family, station number, and isolation order.
(XLSX)

S2 Table Summary of sequencing data in this study. (XLSX)

S3 Table *M*. *kamchatka*- and *M*. *sibericum*-specific genes found in the Mo1-1 genome. (XLSX)
