# Supplementary figures and images for: Isolation of viruses, including mollivirus, with the potential to infect Acanthamoeba from a Japanese warm temperate zone
Source: PLoS One. 2024 Mar 28;19(3):e0301185. doi: 10.1371/journal.pone.0301185 (PMC10977731; doi:10.1371/journal.pone.0301185)

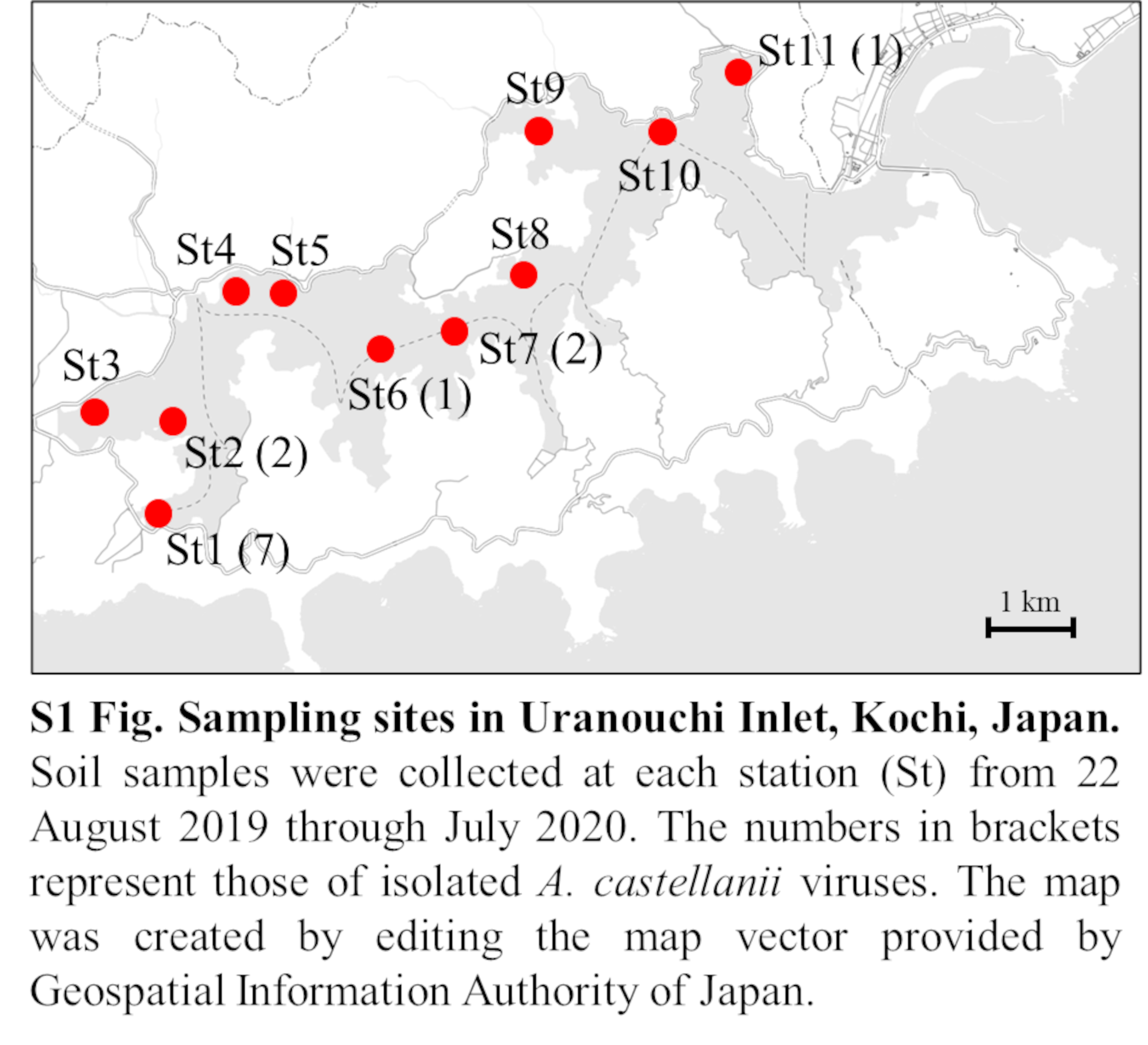

Supplement: S1 Fig — Soil samples were collected at each station (St) from 22 August 2019 through July 2020. The numbers in brackets represent those of isolated A. castellanii viruses. The map was created by editing the map vector provided by Geospatial Information Authority of Japan. (TIF) [file pone.0301185.s001.tif]

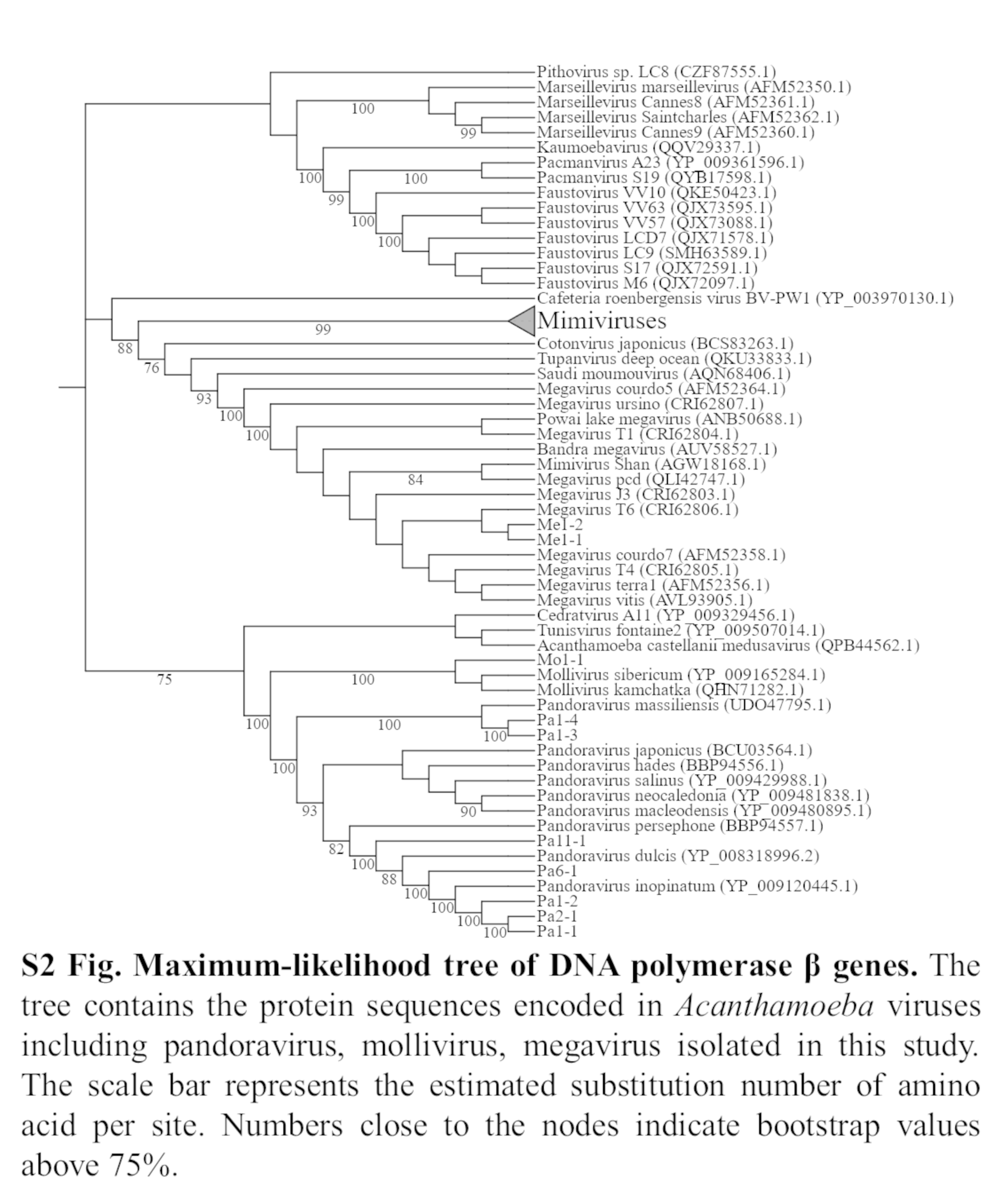

Supplement: S2 Fig — The tree contains the protein sequences encoded in Acanthamoeba viruses, including the pandoraviruses, molliviruses, and megaviruses isolated in this study. The scale bar represents the estimated substitution number of amino acids per site. Numbers close to the nodes indicate bootstrap values above 75%. (TIF) [file pone.0301185.s002.tif]

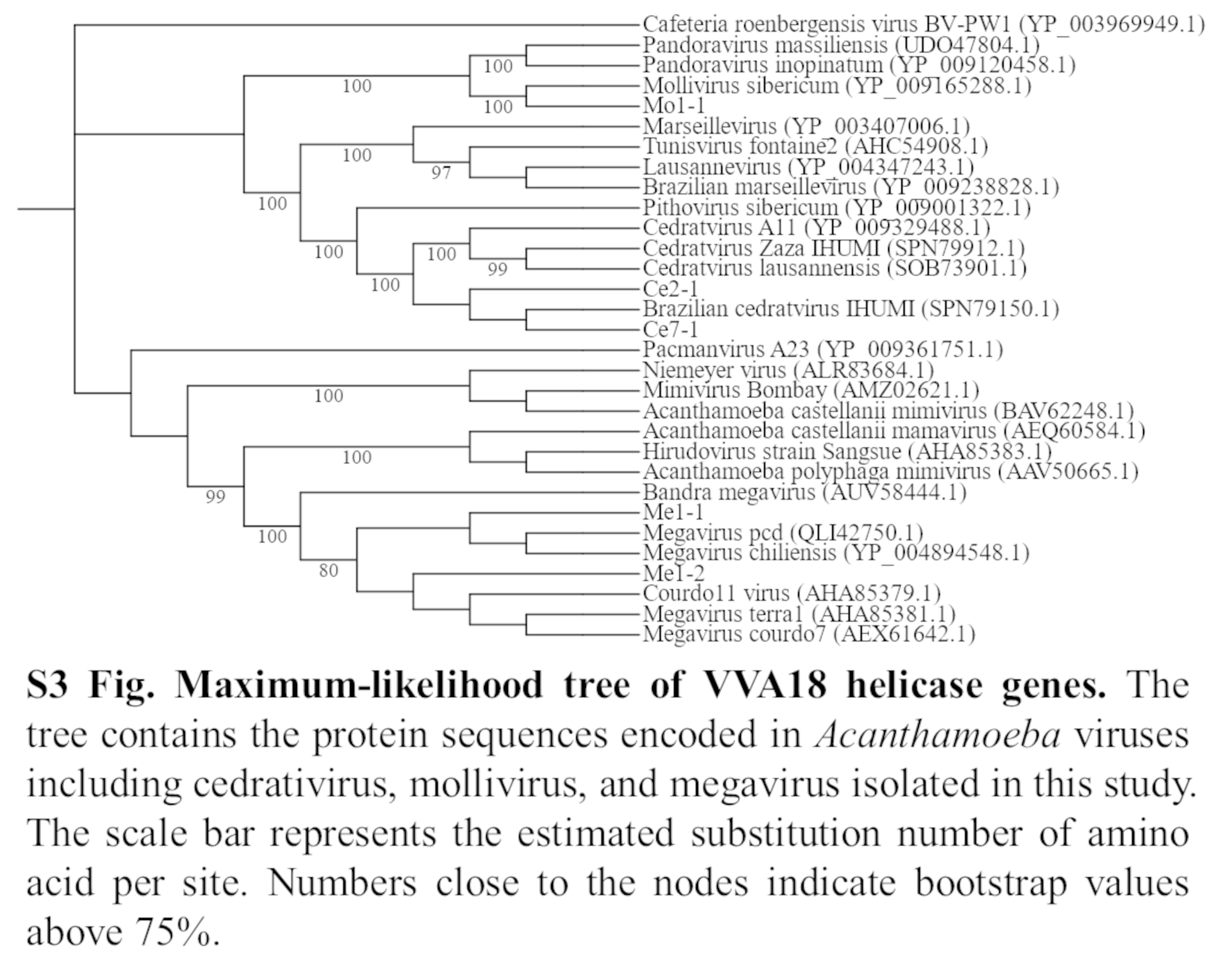

Supplement: S3 Fig — The tree contains the protein sequences encoded in Acanthamoeba viruses, including the cedrativiruses, molliviruses, and megaviruses isolated in this study. The scale bar represents the estimated substitution number of amino acids per site. Numbers close to the nodes indicate bootstrap values above 75%. (TIF) [file pone.0301185.s003.tif]
